# La desigualdad racial de la producción de hortalizas en fresco y flores de corte en Argentina: análisis en el Gran La Plata

**DOI:** 10.18294/sc.2024.4899

**Published:** 2024-12-23

**Authors:** Nuria Caimmi

**Affiliations:** 1 Licenciada en Ciencias Antropológicas. Becaria Doctoral, Consejo Nacional de Investigaciones Científicas y Técnicas, con sede en el Centro de Estudios en Nutrición y Desarrollo Infantil, Buenos Aires, Argentina. nuriacaimmi@gmail.com Consejo Nacional de Investigaciones Científicas y Técnicas Consejo Nacional de Investigaciones Científicas y Técnicas Centro de Estudios en Nutrición y Desarrollo Infantil Buenos Aires Argentina nuriacaimmi@gmail.com

**Keywords:** Alimentación, Racismo, Agricultura, Migrantes, Argentina, Feeding, Racism, Agriculture, Migrants, Argentina

## Abstract

Este trabajo propone analizar la configuración de las desigualdades en uno de los cordones de producción de hortalizas en fresco y flores de corte más importantes de Argentina, localizado en el Gran La Plata, provincia de Buenos Aires. Se trata de una investigación en curso con enfoque antropológico, realizada desde el año 2021, en la que se emplearon técnicas como la observación participante, entrevistas, mapeos alimentarios y etnografía digital. Entre los principales hallazgos destacamos que este cordón productivo posee en su génesis y cotidiano, ciertas dinámicas racistas, que no son únicamente generadas desde la sociedad receptora a la migrante, sino que se propagan por todo el tejido social, incluso ejercidas por los productores migrantes. Las valoraciones positivas de quienes habitan y trabajan allí en torno a los alimentos ultraprocesados están cargadas de la importancia de pertenecer a la sociedad receptora, en paralelo a estrategias de resguardo de preparaciones previas a migrar: la recreación de un “nosotros alimentario” y la proliferación de “legumbrerías” y “comedores paisanos”. Estas transformaciones se enlazan con los cambios productivos que atravesaron las personas migrantes desde economías campesinas en su lugar de origen, a la producción intensiva en el Gran La Plata.

## Abstract (en)

This study aims to analyze the configuration of inequalities in one of Argentina’s most significant production zones for fresh vegetables and cut flowers, located in Greater La Plata, Buenos Aires Province. This ongoing anthropological research, initiated in 2021, employs methods such as participant observation, interviews, food mapping, and digital ethnography. Among the main findings, we highlight that this production zone exhibits, in both its origins and daily operations, certain racist dynamics that are not solely generated by the host society toward migrants but are disseminated throughout the social fabric, including by migrant producers themselves. Positive valuations of ultra-processed foods by those living and working in the area reflect the importance of belonging to the host society, running parallel to strategies for preserving food preparations from before migration. These include the recreation of a “food identity” and the proliferation of “legumbrerías” (legume shops) and “paisano eateries” (restaurants serving traditional food). These transformations are intertwined with the productive changes migrants have experienced, shifting from peasant economies in their places of origin to intensive production in Greater La Plata.

## INTRODUCCIÓN

Decir que el modelo agroalimentario actual es desigual sería, quizás, un oxímoron, siendo que lo que parece tener de alimentario es poco. Particularmente en Argentina, los indicadores alimentarios producidos por el Observatorio de la Deuda Social Argentina^2^, en enero del 2024, muestran cifras alarmantes: el 57,4% de las personas se encuentra por debajo de la línea de pobreza, lo cual arroja cerca de 27 millones de pobres en Argentina^1^. A su vez, la última Encuesta Nacional de Salud y Nutrición de 2019 estima que el 7,9% de menores de 5 años posee baja talla para la edad, y más del 40% de entre 5 a 17 años exceso de peso (dentro de ese total, el 20,4% tiene obesidad). Esto se ve acompañado por las cifras que indican que el quintil más alto de ingresos reporta casi el doble de consumo de frutas que el quintil más bajo de ingresos (45,3% vs. 22,8% respectivamente)^2^. 

Esto da cuenta de la clara desigualdad del sistema alimentario argentino, que se aborda en este trabajo con relación a los procesos y las dinámicas de racialización. La advertencia de que el racismo es una de las modalidades de discriminación más importantes en Argentina^3^, percibido como una problemática que acontece, sobre todo, en otros países, por fuera de Argentina^4^, no hace más que llamar la atención sobre la pregunta por la desigualdad en la esfera alimentaria. Al respecto, Menéndez^5^ alerta que en trabajos epidemiológicos y antropológicos existen ciertos hiatos en las descripciones y análisis de los procesos de salud-enfermedad-atención, como la escasa o nula inclusión del racismo en el estudio y análisis de estos procesos. 

Haciendo eco de estas menciones, este artículo parte de la siguiente pregunta general ¿cómo se expresa la desigualdad racial en el sistema agroalimentario argentino? Siguiendo este interrogante, se recortará su indagatoria en un lugar particular de producción de alimentos, como el cordón productivo ubicado en el Gran La Plata (el cordón fruti-flori-hortícola platense), periurbano productivo localizado en la provincia de Buenos Aires. Es así que el presente trabajo nace con el objetivo de analizar las formas en que se configuran desigualdades raciales en este cordón. Es fundamental señalar que el racismo es pensado como pregunta, como una construcción heurística que motoriza indagar estos procesos en una geografía en especial. 

### Referencias teórico-conceptuales

La alimentación es el proceso por el cual lo necesario para vivir es seleccionado, procesado y entendido, según categorías culturales situadas^6^, siendo que las personas nos alimentamos de nutrientes y sentidos^7^. Más aún, todo análisis del consumo alimentario no puede pensarse por separado del modelo alimentario en su totalidad, es decir, desde el proceso de producción, traslado, distribución y preparación^8^. Contreras^9^ plantea que, a partir de la industrialización y urbanización de las sociedades, la alimentación se ha modificado con el pasaje desde configuraciones regionales, heterogéneas y diversificadas, a otras hiperespecializadas e integradas en una escala global; con la consecuente desaparición de variedades vegetales y animales que antaño habían sido fundamentales en las dietas humanas. Esto ha alterado las relaciones que los grupos habían establecido históricamente con el medioambiente, perdiendo el contacto con el ciclo productivo de los alimentos y acercándolos cada vez más al laboratorio y la industria^10^. Una de las claves de este modelo es la diseminación de nuevas dietas alimentarias, caracterizadas por el aumento de productos ultraprocesados en el mercado, que crean nuevas identidades y patrones de consumo homogéneos^11^, en lo que se ha denominado *macdonalización* alimentaria^12^. 

Sin embargo, siguiendo con Contreras^9^, cuando se habla de la homogeneización de los consumos parece incorporarse solo un aspecto de esa referencia: los productos “disponibles” y no los realmente ingeridos, ni las situaciones que acompañan el consumo, o la identidad de quienes ingieren (o no lo hacen). Cabe señalar que el acceso a los alimentos está organizado socioculturalmente, por lo que el supuesto general de la homogeneización alimentaria debe contrastarse con la persistencia de la desigualdad social en clave de clase, género, edad o, como se trabajará en este artículo, de la pertenencia a colectivos racializados, en el acceso y elección de determinados tipos de alimentos. El último eje es el punto de partida y motor del presente análisis. 

En este marco, es pertinente considerar la categoría “raza”. La noción de razas humanas surgió en el pensamiento científico europeo a finales del siglo XVII y alcanzó una mayor consolidación durante los siglos XVIII y XIX, hasta mediados del siglo XX. Como elabora Quijano^13^, esta noción implicaba una forma particular de conceptualizar la diversidad física, segmentándola y tipificándola arbitrariamente, para luego absolutizar estas diferencias de manera falsa, generando diferencias cualitativas percibidas como excluyentes e incompatibles entre los grupos humanos. Las razas no solo fueron consideradas datos empíricos incuestionables, sino que también se constituyeron como categorías válidas y objetivas de análisis, asumidas como neutrales para clasificar a los seres humanos. Para consolidar esta narrativa sobre el origen humano en las sociedades burguesas del siglo XIX, el discurso evolucionista de las ciencias naturales se cargó de un fuerte componente metaempírico, emocional e ideológico, alineado con los valores de las sociedades coloniales burguesas de la época^14^. Este proceso se dio en el contexto de la expansión imperialista de las potencias europeas, que respondían a las necesidades del capitalismo emergente^13^.

Se ha afirmado científicamente que no existen “razas” en términos biológicos dentro de la especie humana, y que las diferencias raciales, cuando se utilizan para justificar desigualdades y exclusiones, son construcciones sociohistóricas^15^. A pesar de que la “raza” ha sido descartada como categoría científica, sigue siendo una parte esencial de la justificación ideológica de las relaciones socioeconómicas de poder y explotación, por lo cual en este escrito se hablará de procesos de racismo. En este sentido, el racismo no puede entenderse como una simple consecuencia de ideas retrógradas de unos pocos individuos, sino como un fenómeno con alcances estructurales que atraviesa las acciones y pensamientos cotidianos de manera tan sutil que puede incluso pasar desapercibido para los propios sujetos que lo reproducen^16^. Este fenómeno se caracteriza por la creencia de que un grupo humano es intelectual, psicológica o culturalmente inferior a otros, basándose en características visibles en el fenotipo o en la cultura, que se generalizan como marcas distintivas y se interpretan como naturales y hereditarias. 

El racismo, por lo tanto, implica un intento de naturalizar y fijar esas diferencias, y así asegurarlas para siempre: si las diferencias son “culturales”, entonces están abiertas a la modificación y al cambio; pero si son “naturales”, entonces están fuera de la historia, son permanentes y fijas^17^. Este proceso de jerarquización y dominación, se ha reproducido a lo largo de los siglos y sigue siendo una estructura de poder sobre la que se levanta la distinción entre lo “humano” y lo “sub-humano”. Para Franz Fanon, las personas que están por encima de la línea de lo humano son reconocidas socialmente como seres con subjetividad y derechos, mientras que aquellas por debajo de esa línea son despojadas de su humanidad y negadas como sujetos de derechos^18^. De allí que el concepto de “racialización” busca referir a la marcación de las diferencias humanas de acuerdo con los discursos jerárquicos fundados en los encuentros coloniales y en sus legados nacionales^16^. 

Wade^19^ señala que existen posturas encontradas sobre el uso de categorías raciales en análisis sociales de la actualidad. Algunos consideran que su utilización refuerza la noción biológica de la raza, perpetuando así el racismo; mientras que otros defienden su uso como herramienta valiosa para combatir el racismo, al entenderlo como un sistema que, aunque históricamente basado en la idea biológica de la raza, no depende de esta idea, sino que echa luz sobre aquello silenciado. Es en este sentido que aquellos proyectos de integración social, que “no ven la raza” o procuran su “limpieza” por sus implicancias en el pasado, pueden terminar por renovar imaginarios y pactos que aseguran la transmisión o monopolio de cierto capital simbólico racista^20^. En esta línea, Rita Segato^21^^,^^22^ entiende la raza como un signo de una historia de dominación colonial que persiste hasta la actualidad, y que por eso en América Latina son pocos los datos estrictamente raciales, los cuales suelen ser imprecisos, basados en las percepciones de los observadores. También Restrepo y Arias^23^ argumentan que la palabra “raza” fue deliberadamente borrada de los análisis en un esfuerzo por evitar su reproducción, aunque advierten que el problema no se resuelve con una purga generalizada de la palabra, ya que en lugar de ello surgen otros términos que operan como eufemismos, manteniendo intacto el andamiaje ideológico y comportamental sobre el cual se edifica el racismo. 

En este artículo, se retoma esta discusión, argumentando que, para comprender los procesos y efectos del racismo y la racialización, es necesario recuperarlos críticamente, no leídos para denotar la existencia de entidades biológicas desagregadas, sino como fenómenos históricos y sociales de exclusión que construyen esta racialización: las diferencias sociales no pueden ser entendidas sin considerar las diferencias raciales, y el racismo no puede ser visto como una categoría ahistórica e inmutable, sino como un fenómeno que está profundamente imbricado con los contextos particulares en los que se manifiesta^24^.

En el contexto argentino, el racismo es una de las formas más relevantes de discriminación, aunque a menudo se percibe como una problemática ajena, localizada en otros países o regiones^4^. Esto ocurre, en gran parte, porque se reduce el racismo a sus manifestaciones más evidentes, como la discriminación racial, ignorando otras formas de racismo, como la omisión en la “historia oficial” de los despojos, las masacres, los desplazamientos forzados, la esclavitud o la falta de políticas reparadoras^25^. En Argentina, el racismo adquiere una particular dinámica marcada por la invisibilización de ciertos grupos y la construcción de una nación percibida como “blanca”, en contraste con otros discursos que celebraban el mestizaje propio de América Latina^26^. Como señala Gordillo^27^, la “Argentina blanca” ha sido un proyecto de clase que busca presentar el país como predominantemente europeo. Esa narrativa de “blanquedad”^28^, que precisa ser producida y reproducida a nivel micro y en las interacciones cotidianas. Así, los imaginarios racistas, lejos de ser fenómenos aislados o anecdóticos, están profundamente entrelazados con la estructura social y política del país, reflejando y reforzando las jerarquías que definen la pertenencia, la ciudadanía y los derechos en la sociedad argentina.

### Sobre el cordón fruti-flori-hortícola platense

**Figura 1:**
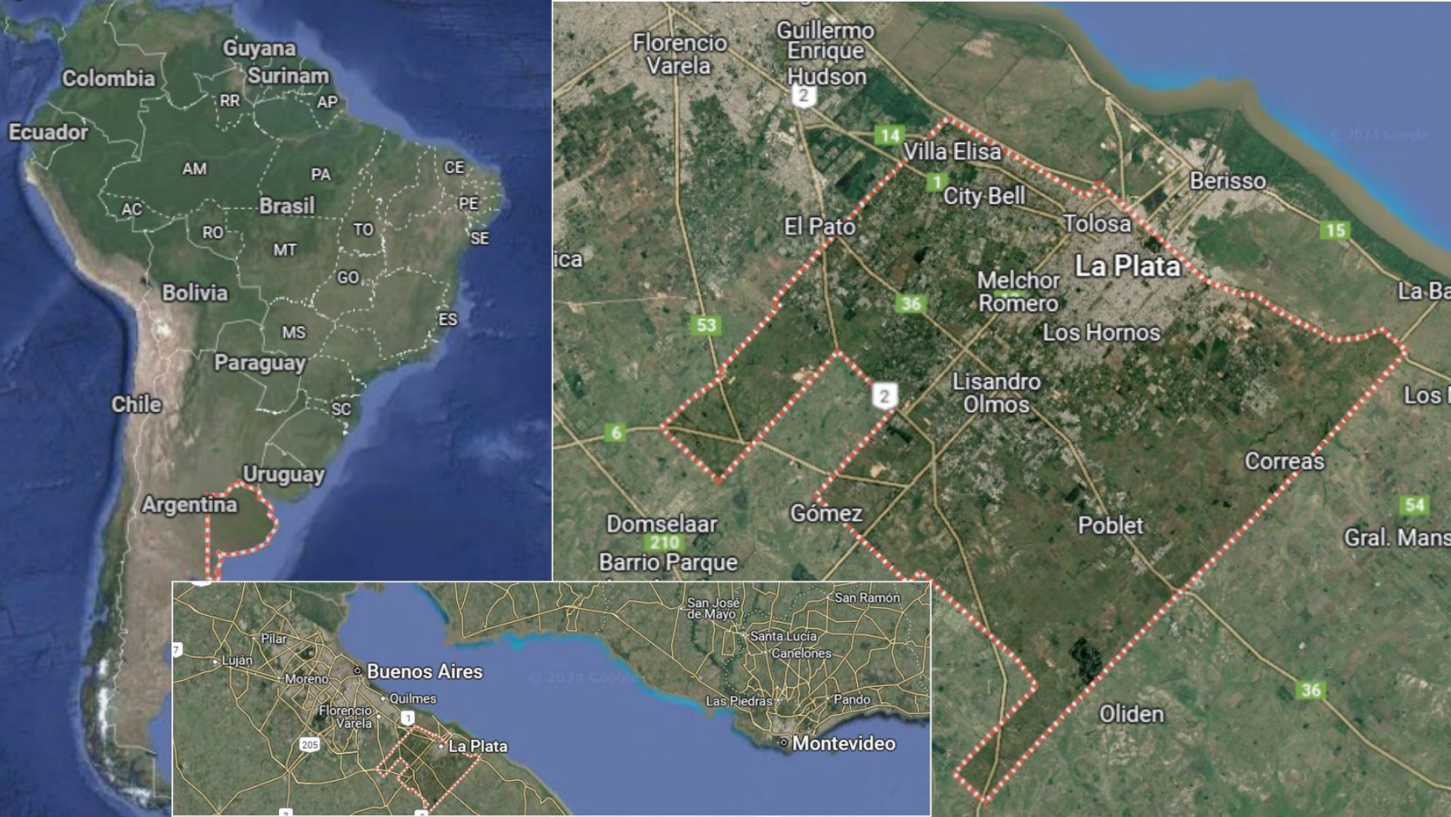
El cordón productivo platense, Gran La Plata, provincia de Buenos Aires, Argentina, 2023-2024.

El cinturón o cordón productivo fruti-flori-hortícola del Gran La Plata (o cordón productivo platense), se localiza en el periurbano de la ciudad de La Plata, provincia de Buenos Aires, Argentina (Figura 1), reconocido por ser el más capitalizado y tecnologizado del país^29^. 

Fuente: Elaboración propia.

Desde fines del siglo XIX, este espacio fue destinado a población proveniente de Italia, España y Portugal^30^^,^^31^, para garantizar la producción de alimentos para la incipiente ciudad de La Plata. Estos migrantes de ultramar prevalecieron en la actividad hasta mitad del siglo XX, cuando se produjo un recambio poblacional, que dio paso a migrantes del norte del país y, fundamentalmente, de Bolivia. Durante la década de 1980 y 1990, se expandió el proceso denominado “bolivianización de la horticultura”^32^^,^^33^, por el cual, a semejanza de otros cinturones verdes del país, las personas migrantes bolivianas se constituyeron en el actor mayoritario, no solo en tareas productivas, sino cada vez más en tareas logísticas y comerciales. 

La estructura productiva del lugar está condicionada por la transformación que atravesó en las décadas de 1980 y 1990 con la instalación y consolidación de un modelo basado en el uso de grandes dosis de insumos externos y una fuerte producción bajo plástico, con la tecnología de invernadero^29^. Por eso, en una sola generación, quienes llegaron a la Argentina atravesaron un cambio abrupto en las formas de producir. Si en Bolivia, las explotaciones eran de tipo campesinas para el consumo doméstico, en Argentina, los preceptos de la revolución verde estructuraron la matriz del cordón productivo, con el uso intensivo de invernaderos y de paquetes tecnológicos. Además, en la trayectoria de migración a Argentina, las personas migrantes entraron en contacto con una configuración nacional distinta a la de origen, con imaginarios de homogeneidad e intolerancia a las diferencias, que les permitiera alejarse del ideal europeo^26^. 

García y Quaranta^34^, a partir de una investigación con imágenes satelitales, alcanzan el dato de 3.800 establecimientos hortícolas y 8.612 de hectáreas hortícolas. Estos establecimientos hortícolas se denominan habitualmente “quintas”, con una extensión de entre 1 a 4 hectáreas. Las unidades productivas albergan en su interior las unidades domésticas, dado que los hogares se ubican dentro del predio productivo. Estos hogares están compuestos por personas asociadas entre sí por distintos grados de parentesco, habitualmente, una familia nuclear compuesta por madre, padre e hijos. Sin embargo, se registran también familias monoparentales (mujeres madres), la presencia de abuelas y abuelos (que en algunos casos ofician de padres con sus nietos), así como tíos y primos en las viviendas. Las mujeres productoras realizan casi con exclusividad los trabajos domésticos y de cuidado, a la par que trabajan junto al varón en la producción hortícola^35^.

Con relación a la tenencia de la tierra, la figura prevalente es el arrendamiento, aunque la informalidad de estos acuerdos con los dueños de la tierra implica que sea habitual el desalojo o el aumento del valor por fuera de los acuerdos establecidos, lo cual expulsa a las familias a irse del predio y comenzar de nuevo en otro. Además, las condiciones de los contratos de alquiler mayormente no proveen de casa a los arrendatarios ni les permiten la construcción de infraestructura o, en caso de que lo hagan, no es reconocida la inversión. Esto lleva a la construcción de casas fácilmente desmontables de madera, chapa y nailon, con un pozo ciego para evacuación y otro para la bomba, que garantice la provisión de agua para consumo y riego^36^. 

El tipo de producción predominante es conocido como “convencional”, bajo la utilización del paquete tecnológico. Quienes producen de esta forma, cuentan con poca diversidad en sus producciones, dado que se destinan al mercado. Las jornadas de trabajo son de entre 8 y 16 horas (según temporada invernal o estival) y las labores realizadas bajo cubierta o campo abierto son extremadamente duras. Además, la exposición a tóxicos de los insumos que utilizan es permanente, con graves efectos en su salud. 

**Figura 2:**
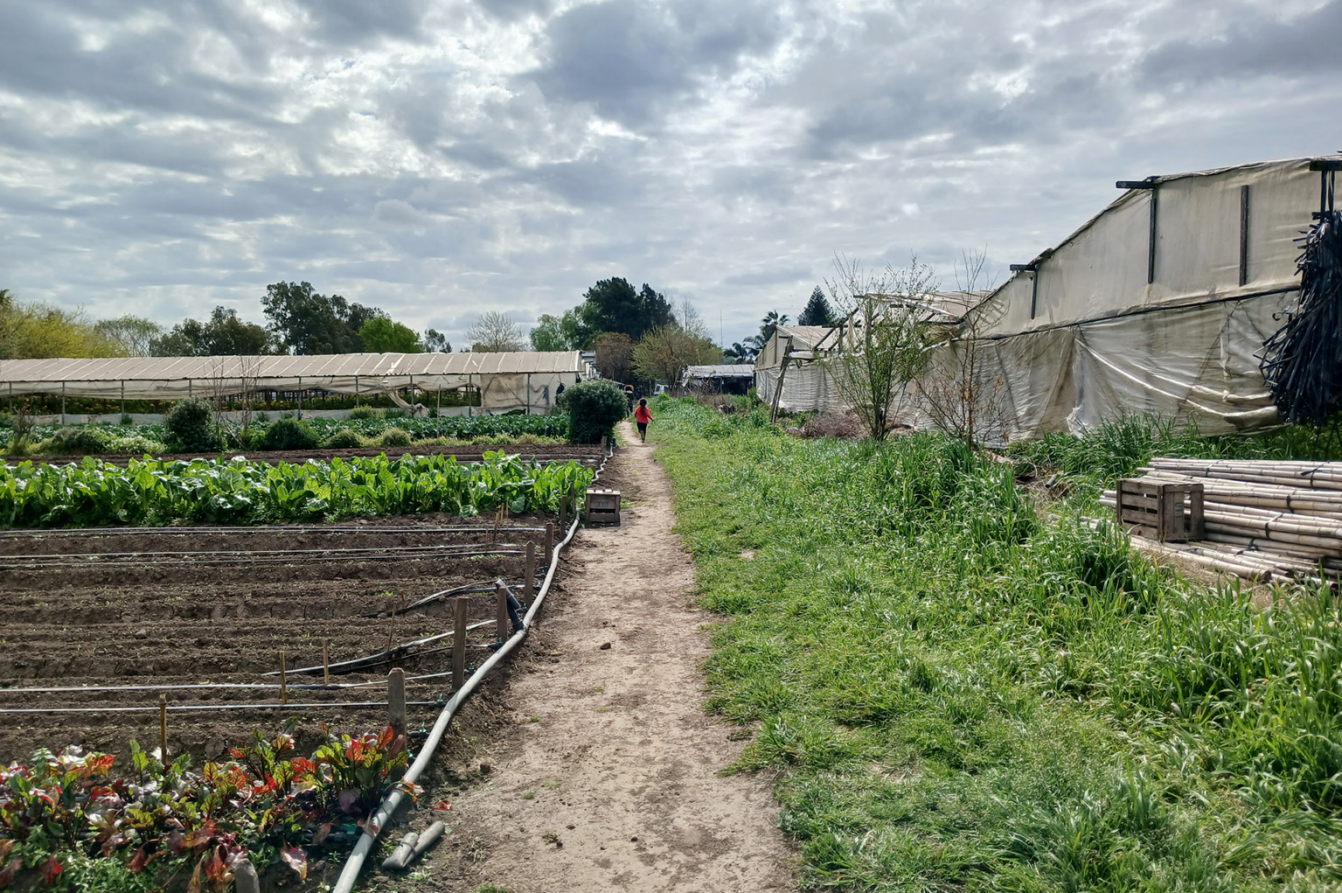
Quinta productiva en el cordón platense, Gran La Plata, provincia de Buenos Aires, Argentina. Septiembre, 2023.

Distintos estudios dan cuenta del grado de exclusión y pobreza en que viven los productores, dado que sus ingresos no permiten acceder a los bienes de consumo básicos estipulados por la sociedad actual para gozar de un buen vivir^37^^,^^38^. Por eso, estas familias llevan a cabo estrategias domésticas tendientes a su reproducción, que consisten en la intensificación de la mano de obra familiar, la contracción del consumo y la maximización de bienes de uso^29^. Aunque en algunos casos se documenta un ascenso social, esto no implica que se reviertan las condiciones de informalidad que caracterizan al sector (Figura 2).

Fuente: Fotografía tomada por la autora durante el trabajo de campo.

## ESTRATEGIAS METODOLÓGICAS

Este estudio parte de una perspectiva antropológica y etnográfica, entendida como una práctica de investigación que busca comprender un segmento del mundo social a través de un análisis centrado en las perspectivas de los actores nativos^39^ y en su interacción con contextos más amplios^40^. El área de investigación es el cordón fruti-hortícola de La Plata, donde se realizó trabajo de campo entre el año 2021 y 2024 con productores de hortalizas, flores y frutas. Para el presente estudio, se priorizan los registros y observaciones de los últimos dos años (2023 y 2024), ya que la proximidad con los participantes ha facilitado un mayor nivel de profundidad en los intercambios.

Las técnicas de investigación empleadas se centraron, principalmente, en entrevistas semiestructuradas y observación participante, debido a que muchas de las prácticas relacionadas con la alimentación no se verbalizan explícitamente en los discursos espontáneos, lo que permite registrar las tensiones entre lo que las personas dicen y lo que hacen^41^. Además, desde 2021 se realizó una metodología de “mapeos alimentarios” en el marco de talleres realizados entre equipos de extensión de la Universidad de Buenos Aires y productores del cordón^42^. Por último, se resalta la utilización de la técnica de la etnografía virtual mediante tecnologías digitales^43^.

La selección de los participantes se realizó dentro de una muestra exploratoria más amplia, en la que se priorizó a migrantes de procedencia boliviana que vivieran y trabajaran en la horticultura o floricultura en el cordón platense. 

En el caso de las entrevistas, se seleccionó a aquellas participantes con mayor afinidad a la temática de pesquisa. Se entrevistó a cinco mujeres migrantes, de entre 42 y 49 años:

- Trifona (45 años), migrante boliviana (Tarija), productora de hortalizas agroecológicas.
- Elodia (49 años), migrante boliviana (Chuquisaca), productora de hortalizas agroecológicas.
- Rosa (46 años), migrante boliviana (Tarija), productora de hortalizas, huevos y frutales agroecológicos.
- Rosmery (42 años), migrante jujeña, productora de hortalizas y verdulera.
- Tomasa (42 años), migrante boliviana (Tarija), productora de hortalizas y empleada doméstica.

Las entrevistas se llevaron a cabo en las quintas de las participantes o en la sede de una de las organizaciones del sector, ubicada en la localidad de Lisandro Olmos. La duración de las entrevistas varió entre 40 minutos y una hora y diez minutos, grabadas en dispositivos móviles, mientras se tomaban notas en un cuaderno sobre aspectos contextuales, cambios en el tono de voz, silencios, preguntas y otros elementos relevantes. En algunos casos, la cercanía establecida con las participantes permitió que se compartieran aspectos de sus historias de vida que no fueron abordados en las entrevistas formales, sino en diálogos informales, los cuales fueron transcritos de manera fiel. 

En cuanto a la observación participante, se generó un amplio material a través de conversaciones informales y registros de campo, realizados en diversos contextos: quintas (en espacios productivos y domésticos), sedes de organizaciones gremiales de productores, comedores, legumbrerías, otros comercios, remiserías y vehículos de remises. Un total de 13 personas de entre 21 y 49 años fueron claves en este estudio, todos migrantes procedentes de Bolivia, que trabajaban en la horticultura y la floricultura.

La técnica de “mapeos alimentarios” fue realizada en el marco de talleres llevados adelante por equipos de extensión de la Universidad de Buenos Aires en articulación con productoras del cordón. Estos talleres, se realizaron en las bases de organizaciones sociales conformadas por pequeños productores del sector (dedicados a la horticultura y la floricultura), en los cuales se abordaron cuestiones nutricionales, sociales, culturales y culinarias en relación con la alimentación. Como parte de las estrategias pedagógicas desplegadas en estos encuentros, se construyó la metodología de mapeos alimentarios, que también constituye una técnica de investigación. Estos mapeos, consistieron en la representación gráfica de platos de comida y bebidas de “antes” y “ahora” elaborados por parte de los productores de alimentos que asistían a los talleres. Algunas de las personas entrevistadas para este estudio formaron parte de la coordinación de estos talleres, mientras que otros fueron participantes. Para este estudio, se recuperaron 15 mapeos realizados por productores flori-hortícolas de entre 9 y 70 años, durante los años 2022-2024. Tanto niños, jóvenes como adultos participaron de estas instancias, como parte de su formación alimentaria en los talleres mencionados. La sistematización de estos mapeos se realizó mediante la clasificación de los alimentos y bebidas representados en los momentos del “antes” y el “presente”^42^.

Por último, se trabajó con dos técnicas de etnografía digital: el análisis de un grupo de Facebook denominado “Quinteros La Plata” y el registro de estados de WhatsApp relacionados con alimentos. En cuanto al procedimiento, la información recuperada en Facebook se seleccionó de acuerdo con el orden de relevancia determinado por el nivel de interacción de los miembros del grupo, lo que implicó un enfoque exploratorio y no exhaustivo. En el caso de los estados de WhatsApp, estos se han registrado con personas previamente informadas sobre este estudio, con quienes se ha mantenido un diálogo sobre las imágenes compartidas. 

Todo el proceso de registro de información se documentó a través de cuadernos de campo, grabaciones y fotografías, así como mediante la elaboración de fichas temáticas y planillas de observación. Las notas de campo se tomaron de forma simultánea al diálogo, en un cuaderno de papel o en un dispositivo digital y, en algunos casos, se completaron posteriormente al intercambio. Estas notas incluyeron descripciones detalladas de las observaciones y transcripciones de las entrevistas, en las que se incorporó también información contextual relevante. Las principales dimensiones que orientaron las entrevistas, diálogos informales y observaciones fueron los procesos alimentarios, particularmente aquellos relacionados con situaciones y experiencias de discriminación, exclusión, alteridad, prestigio, orgullo y deseo, que permitieran profundizar en las valoraciones sobre la alimentación, tanto en espacios privados como públicos, a partir de la experiencia migratoria, e indagar en los cambios diacrónicos desde la llegada a La Plata hasta el momento de realización de la pesquisa. 

La sistematización y análisis de estos registros permitió ajustar los instrumentos y comprobar las hipótesis emergentes. Este enfoque sistemático facilitó la construcción de los datos empíricos, la interrogación del material recolectado y la constante reflexión teórica, a fin de generar nuevas interpretaciones y relaciones a partir de los hallazgos. 

Todos los participantes de esta investigación fueron informados sobre el estudio en general. En todos los casos, se aseguró de explicitar claramente los objetivos de la investigación, las estrategias metodológicas, el tratamiento de la información, su destino y confidencialidad, y el compromiso de mantener el anonimato de los participantes durante todas las fases del estudio, alterando los nombres originales de personas y lugares por otros ficticios para preservar la confidencialidad. 

## RESULTADOS

### El racismo en la génesis y el cotidiano del sector

Al transitar por el cordón platense, en algunas ocasiones, se escuchó a actores, que no eran migrantes ni productores, denominar el lugar como “*bolilandia*” o “*paisanolandia*”. ¿Puede aseverarse que esta categoría posee una valorización positiva o negativa *a priori*? Si bien no puede aseverarse que esta categoría posee una valorización positiva o negativa *a priori*, sí alude, al menos, a un proceso de homogeneización al interior de los grupos migrantes. Diversos estudios^26^^,^^44^ han dado cuenta de que la categoría de “boliviano” es utilizada comúnmente en lugares para designar no solo a las personas que nacieron en Bolivia, sino también a sus hijos, aunque sean legalmente argentinos. Esto fue referido en diálogos con interlocutoras, especialmente con Gilda (29 años) y su madre, Trifona (45 años):

Trifona: *Acá no se ven argentinos, ¿vos viste?*

Gilda: *Acá hay solo bolivianos y japoneses, por ahí en los barrios hay argentinos, pero no nos cruzamos.*

Trifona: *Aunque vaya a la ciudad* [de La Plata]*, allá también es toda paisanada.* (Registro de campo, marzo 2024)

Acompañando a alguna de ellas a esa ciudad, se encontró que muchos de los circuitos que conectan a las productoras con la urbe se vinculan con servicios de asistencia social, a los que efectivamente acuden migrantes, también de Bolivia. Ni la caracterización de “*bolilandia*” ni la escasa presencia argentina en estos circuitos denominan de antemano un escenario de discriminación, pero sí apuntan cierto proceso segregación espacial y construcción de la alteridad. 

En este sentido, como resaltan Chiriguini^45^ y Chiriguini y Mancusi^46^, todo proceso de reconocimiento de la diversidad, reclama una contextualización de esas diferencias delimitadas dentro de procesos históricos de dominación, explotación y exclusión de las realidades sociales concretas donde se producen. Toda relación de alteridad, como la que se da en el encuentro entre migrantes y la sociedad receptora, está atravesada por el etnocentrismo, actitud de un grupo social que considera su cultura como el estándar de referencia, juzgando otras culturas como “raras”, “anormales” o “patológicas”. Esto se manifiesta cuando un grupo niega o desprecia las diferencias y no acepta la diversidad, expresándose mediante ignorancia, desprecio o lenguaje despectivo hacia los demás^46^. En los viajes hacia el cordón platense, era común tomar remises locales para acceder a sitios difíciles de llegar. En las charlas con conductores, uno de ellos, mencionó lo siguiente: “Él dijo que el 80 por ciento de sus clientes son bolivianos, que los criollos “así como vos” son los menos” (Registro de campo, junio 2023). Además de esta caracterización, los choferes solían señalar con sorpresa las elecciones que las personas hacían:

*Contó del ascenso social de los productores, que cuando consiguen dinero después de unos años de trabajar “Ya no tocan más la tierra”, arriendan o contratan. Habló de las camionetas que se compran, “siempre carísimas”, en alusión a, en vez de comprar tierra propia, preferían algo así, “ostentoso”.* (Registro de campo, julio 2022)

Incluso, cuando desde la ventanilla se veía un predio y un grupo de personas, que no expresaba más información que la descrita, se escuchaba por parte de conductores: “*No sé por qué los bolivianos viven así, les debe gustar, tener la casa con chapas, que se te vuele todo, no lo entiendo la verdad*” (Registro de campo, junio de 2023). Más allá de la estigmatización de estos recortes, que desconocen la realidad de quienes habitan en el cordón (sin posibilidad de acceder a tierra y de construir viviendas, junto con la importancia de los vehículos para transportar alimentos y desplazarse), interesa detenerse en la caracterización de estos relatos, delimitando una otredad marcada por la incomprensión por parte de personas no migrantes ni productoras, del comportamiento de los que sí. En este sentido, si bien la migración boliviana puede rastrearse cuarenta años atrás, lo recuperado denota que aún permanece en conflicto con los habitantes locales e incluso imposibilita que los migrantes se apropien del espacio: por ejemplo, Rubinstein y Lemmi^47^ encontraron en su trabajo una muy baja consideración de los y las productoras migrantes como parte de la historia de las localidades del cordón platense. 

Lejos de ser únicamente expresiones desvalorizantes, muchas veces la marcación de la diferencia aludía a caracterizaciones positivas “*Los bolivianos de chiquitos saben trabajar la tierra, de sol a sol los ves ahí, aunque llueva, si hace frío se hace una fogatita, yo no sé cómo hacen*” (Registro de campo, septiembre 2022). Esto mismo fue recuperado en el trabajo de etnografía virtual. Allí, una publicación en Facebook con muchas interacciones recitaba “*Necesito gente para campo y invernadero, cualquier cosa hablarme al privado, y con preferencia bolivianos*” (Publicación, 28 de diciembre de 2023), o “*Necesito una familia boliviana para trabajar en verdura”*, la cual recibió un comentario que preguntaba por qué la nacionalidad y recibió como respuesta: *“porque los argentinos son vagos*”. A continuación, una serie de comentarios expresaban “*porque los argentinos son vagos*”, “*cuando no queramos trabajar más que van a hacer los argentinos*”, “*hay argentinos que también trabajan aunque son los menos*” (Publicación, 16 de junio de 2024). Más allá del emisor de estas publicaciones, lo que sobresale aquí es la asociación entre nacionalidad y una disposición a cierto tipo de trabajo agrícola. Sin embargo, esta culturalización de la dota laboral reclama un señalamiento fundamental: no son las características culturales las que explican la masiva presencia de bolivianos en esta actividad económica, sino que, teniendo en cuenta un origen vulnerable y su condición migrante, pasan a ser protagonistas porque están dispuestos a aceptar retornos y niveles de acumulación menores por los recursos que ponen en juego en el proceso productivo^48^. 

Al establecer relaciones de confianza con productoras, se encontró en sus historias migratorias desde Bolivia y/o en ocasiones desde el noroeste de Argentina, el abuso, el acoso y la explotación desde niñas. En el caso de Elodia (49 años), ella fue buscada a los 12 años a su casa en Chuquisaca (centro este de Bolivia), por una mujer que prometía contratarla en tareas de cocina; promesa que fue una estafa, dado que terminó encerrándola meses en sus fincas para producción intensiva de hortalizas, sin paga, junto con 30 niños de entre 9 y 15 años (los cuales gran parte no hablaban en español, sino en quechua). Otra de estas mujeres, doña Rosa (46 años), contó que a los 8 años había viajado con su padre desde Tarija (sur boliviano) a Jujuy para trabajar en la cosecha de la caña, donde sufrió muchos años violencia física por parte del patrón de la finca, hasta que a los 16 años pudo escaparse para volver a su tierra. También era de Jujuy el relato de Rosmery (42 años) quien, desde niña, junto a sus ocho hermanos, era jornalera en el tabaco. Sus padres, ambos analfabetos, eran cotidianamente abusados por los patrones, que pagaban solo la subsistencia para que la familia no muriera. 

Estas historias lejos están de agotar la complejidad de trayectorias de vida de las y los productores, ni pretenden ser un listado de las vivencias de quienes hoy trabajan produciendo alimentos en el cordón platense. Pero sí buscan apuntar que parte de la génesis de este espacio productivo ha sido traccionada por escenas en las que las relaciones de explotación y abuso en el derrotero hasta llegar a La Plata, son conformantes. Además, estos relatos permitían trazar un puente con el presente en el cordón platense. 

Esta charla entre Elodia y Tomasa (42 años), es significativa.

Elodia: *Sí, sigue pasando. Es conocido que algunas quintas traen menores de edad de Bolivia o del norte, no sé qué les prometen, pero los traen y los encierran para trabajar.*

Tomasa: *Sí, hay situaciones así en las quintas, quienes estamos acá conocemos.*

Elodia: *Hace dos años estuve con dos chicas en mi casa, ni 15 años, las ayudé porque las trajeron a trabajar, no les pagaban y de una de ellas abusaron sexualmente, el patrón tuvo un hijo con ella*. (Registro de campo, febrero 2024)

Esto ha sido una constante en las charlas más privadas con productores y productoras del lugar, enunciando escenas de encierro, maltrato físico, violencia sexual y psicológica para asegurar la permanencia de las personas como jornaleros o peones en las quintas del cordón. Por ejemplo, Rosa (46 años), la única de todas que tiene tierra, reconocía que, aun así, una de las cuestiones más difíciles de las quintas era vivir con los patrones. Si bien diferenciaba entre buenos y malos, en el caso de estos últimos las condenas pueden ser simbólicas, psicológicas y físicas. “*No te deja que nadie venga a tu casa, no podés tener gallinas ni animales, mucho menos plantar lo que quieras, pero lo peor es que te maltrata, te maltrata por ser boliviano, cree que valemos menos*” (Entrevista Rosa, septiembre 2023). 

Lejos de ser esto un señalamiento exclusivo hacia argentinos o hijos de italianos, una de ellas marcaba que: “*Yo entré a trabajar con el paisano como porcentajera* [que cobra un porcentaje de la ganancia obtenida de la producción], *después salí a alquilar en otro lugar, porque el paisano te abusa a veces más, se queja, se olvida de que es paisano también, que somos hermanos*” (Entrevista Trifona, marzo 2024). Este último fragmento, relacionado con el “abuso” en situaciones laborales, abona al debate sobre la relación entre las nociones de trabajo y explotación laboral^49^. Si el trabajo refiere a las relaciones laborales voluntarias dentro de modalidades legales y aceptadas del capitalismo, junto con prácticas laborales fuera de la legalidad, pero socialmente toleradas (salarios bajos, largas jornadas, falta de descansos, o condiciones riesgosas); la “explotación laboral” describiría prácticas abusivas y naturalizadas, raramente denunciadas, que reflejan la desigualdad inherente a las relaciones laborales. Sin embargo, la distinción entre trabajo y explotación resulta laxa, dado que en ambas la coacción económica y extraeconómica siguen lo suficientemente separadas como para sostenerse dentro de una legalidad normativa, y la legitimidad y tolerancia social^49^. Además, el fragmento resalta que el abuso se da entre paisanos, lo cual puede ser explorado bajo la noción de redes migrantes horizontales y verticales apuntadas por Pedone^50^ para pensar procesos migratorios transnacionales. Con relación al último tipo de redes verticales, la autora sostiene que los recursos sociales que circulan por estas relaciones no excluyen la aparición de actores que detentan el poder, vínculos verticales que otorgan una jerarquía entre sus miembros. Esto se expresa claramente en la modelización analítica construida por Benencia^32^^,^^51^, para representar procesos de ascenso social y contratación entre migrantes bolivianos hortícolas denominado “escalera boliviana”, modelo que permite analizar procesos de contratación y explotación entre paisanos en la horticultura. 

El trabajo etnográfico también permitió recuperar intervenciones por parte de agentes institucionales en las quintas, que ingresaban a los predios para penalizar que los niños y las niñas estuvieran junto a las personas adultas en el trabajo productivo. Ellas explicaban que “*uno lleva al chico porque muchas veces dejarlos solos en las casillas es peor, es peligroso, ha pasado de chicos que se incendian por las chapas, yo prefiero tenerlo conmigo cerca*” (Registro de campo, febrero 2024). En alusión a la ilegalidad del trabajo infantil, estos agentes indicaban que debían pagar un dinero para no ser penalizadas legalmente.

La estigmatización se expresaba también en instituciones de salud. Se registró la afluencia de muchos productores al doctor “La Viña” que atendía en su consultorio privado, casi exclusivamente a productores migrantes. Al preguntar los motivos, compartían que “*te da la pastilla el mismo día*”, “*no te hace recetas ni te da vueltas*” así como “*porque en la salita los tratan mal a los paisanos, o no los quieren atender por ser de Bolivia*” (Registro de campo, diciembre 2023).

En estos pasajes, a la noción de boliviandad se anuda una inferioridad que roza lo no humano y, por ende, habilita el trato deshumanizante. Sin embargo, como apuntan estos recortes, no implica una lectura direccional desde argentinos o italianos hacia bolivianos y bolivianas, sino que el entramado se difuma cuando los paisanos *dejan* o se *olvidan* que lo son y también ejercen estos maltratos. Para esto, se recupera lo elaborado al comenzar este escrito con relación a las formas particulares que adquiere el racismo en Argentina, ligado a cierta narrativa nacional de blanquedad. En el desarrollo y consolidación del Estado-Nación se trabajó en favor de homogeneizar la diversidad, se modeló al sujeto nacional sobre la base de un perfil europeizante, despojando toda particularidad nativa. En esa búsqueda de la configuración de un sustrato nacional homogéneo por parte los pensadores y políticos que definieron el ideario nacional en sus orígenes, también quedaron invisibilizadas las particularidades de aquellos individuos y grupos migrantes^48^. Chiriguini^45^ distingue diversos momentos en la conformación de imaginarios racistas sobre la alteridad en el país, destacando que entre las décadas de 1930 y 1950, los prejuicios raciales se centraron en las grandes migraciones internas impulsadas por la crisis económica de 1930. Este fenómeno generó un sentimiento leído como “invasión”, ya que sectores populares comenzaron a ocupar espacios anteriormente vedados a las clases más desfavorecidas, lo que alimentó la construcción social de un “otro” percibido como una amenaza al orden establecido. Para esta autora, en la actualidad, las representaciones racistas continúan descalificando a los migrantes, particularmente, aquellos provenientes de países limítrofes desde lecturas xenófobas y racistas. Estos estereotipos, que vinculan a estos grupos con características como la lentitud, la pobreza y la falta de higiene, contribuyen a la construcción de una imagen negativa generalizada, que naturaliza y esencializa estos rasgos como inherentes^46^. En este movimiento, Tineo Durán, recupera que, en ciertas construcciones de los migrantes como víctimas, se recrea e instrumentaliza una idea de “duelo migrante” que termina por incurrir en formatos de salvación, caridad o culpa que pautan las relaciones de superioridad blanca^20^. 

En este estudio, además, las historias de vida registradas se caracterizan por ser de mujeres, expresando relaciones de desigualdad en las que se interceptan la pertenencia de clase, género y lecturas racializadas, las cuales se retroalimentan, incrementando las condiciones de vulnerabilidad. En este sentido, debemos resaltar lo que apuntan distintos estudios previos^36^^,^^52^^,^^53^, respecto a que el trabajo productivo de las mujeres en este contexto es considerado como ayuda, mientras que el reproductivo y de cuidados, naturalizados y desvalorizados como tareas propiamente femeninas. 

Estas historias de discriminación poseen un contrapunto que interesa señalar. Lejos de implicar un proceso de borramiento total de “lo paisano”, de una década a esta parte dos procesos parecen tensionar o resignificar este estigma. Por un lado, el aumento de organizaciones sociales y gremiales en el cordón hortícola platense, representativas de productores de alimentos, que han revalorizado o reorientado su praxis en relación con la cuestión étnica y migratoria. Las mujeres con las que se trabajó señalaron constantemente que el aprendizaje de derechos fue resultado de su participación en organizaciones. 

**Figura 3:**
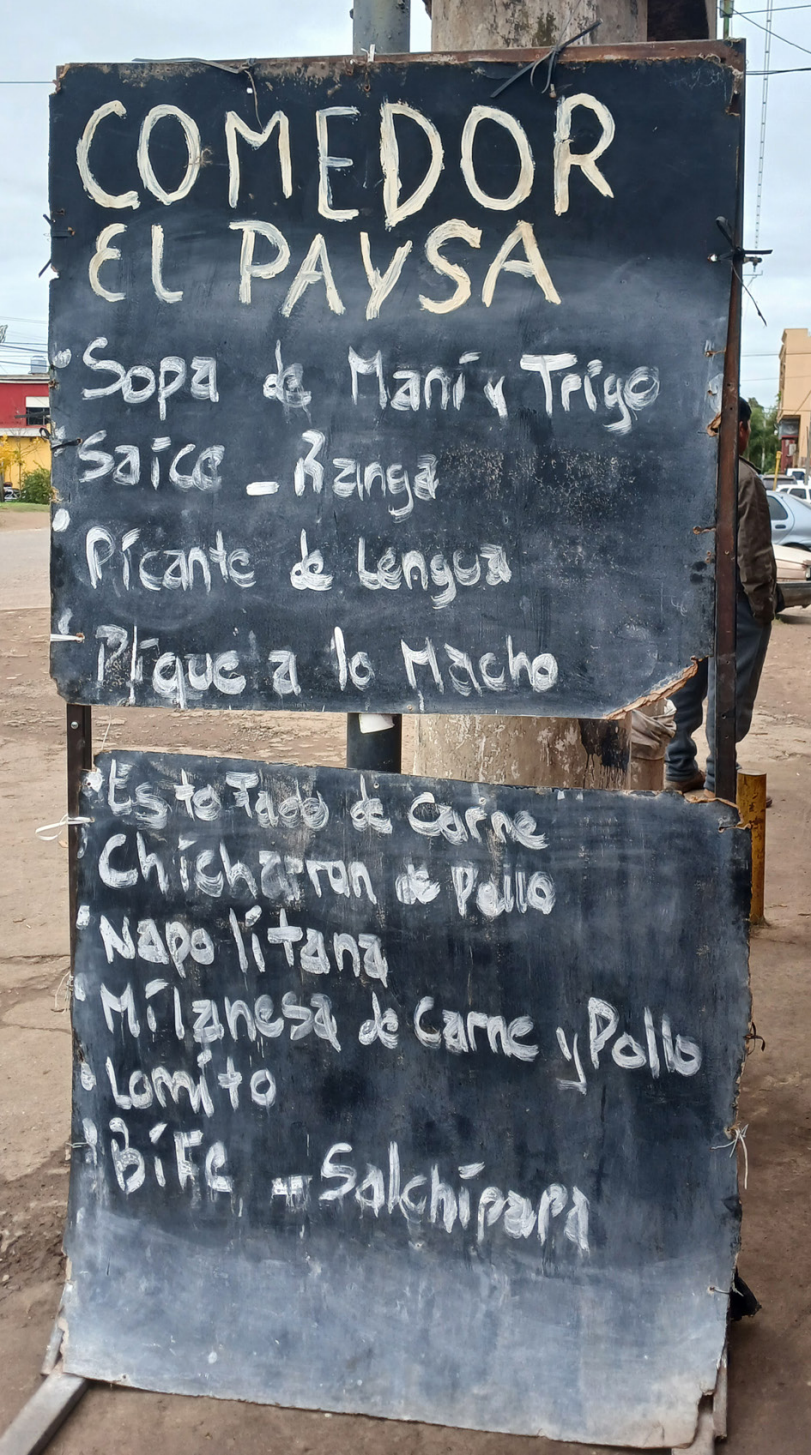
Menú de comidas en un “comedor paisano”. Lisandro Olmos, La Plata, provincia de Buenos Aires, Argentina. Noviembre, 2023.

Por otro lado, han escalado notablemente las “legumbrerías”, en las que se venden granos, semillas, especias (incluso productos secos traídos de Bolivia, como habas, papa lisa, harina de maíz), así como productos de cotillón o repostería; y los “comedores paisanos” al decir de los locales, en los que se sirven preparaciones típicas bolivianas (Figura 3). En el trabajo de campo, se encontró que quienes asisten son personas bolivianas y sus familias, así como obreros o camioneros. Gilda (29 años), trabajó desde los 15 años en un comedor, por lo que pudo documentar la incorporación progresiva de “*comida argentina*” (en sus términos), como milanesa, pizza o matambre. “*Al mediodía suelen ser familias, por la cena, a eso de las 6, son todos obreros o camioneros, no necesariamente paisanos*” (Registro de campo, abril 2024). Por su parte, Tomasa (42 años), expresaba la explosión de comedores en los últimos años. 

Fuente: Fotografía tomada por la autora durante el trabajo de campo.

*Cuando yo llegué, hace casi 30 años ya, casi no había comedores de comida de allá, mucho menos legumbrerías, entonces si querías algo te lo tenían que traer en algún camión, o si algún familiar venía de visita, otros viajaban al mercado de Liniers. Ahora está lleno, los paisanos fueron abriendo.* (Entrevista Tomasa, abril 2024)

Para Waisman^54^, y Waisman y Rispoli^55^, el contexto social del cordón platense, atravesado por la xenofobia y la discriminación actuó como catalizador en la configuración de lazos comunitarios, desplegados para eludir los efectos negativos de la discriminación. “A mayor sospecha, violencia, estigmatización, mayor confianza en los que comparten un mismo origen”^55^. Las autoras señalan que la configuración de un “nosotros” que se encuentra entre los migrantes bolivianos del cordón -que en nuestro trabajo se expresa mediante organizaciones gremiales de productores, legumbrerías y comedores- debe buscarse en la coyuntura crítica de la década de 1990 (por la irregularidad de su condición ciudadana y el progresivo proceso de segmentación de la estructura social hortícola), que se expresó en la creación de espacios que las autoras denominan “enclaves”, lugares de socialización. 

Pizarro^56^ advierte que una interpretación que se limite a enfatizar el rol adaptativo de estos enclaves para su integración a la sociedad receptora resulta insuficiente para dar cuenta de los procesos de discriminación vinculados con la migración. Las irrupciones mencionadas, más que adaptación, condensan procesos activos de existencia en aquel lugar, resaltando la capacidad de los migrantes de gestionar situaciones complejas en contextos de incertidumbre^57^. 

### Transformaciones alimentarias en el cordón productivo platense

**Figura 4:**
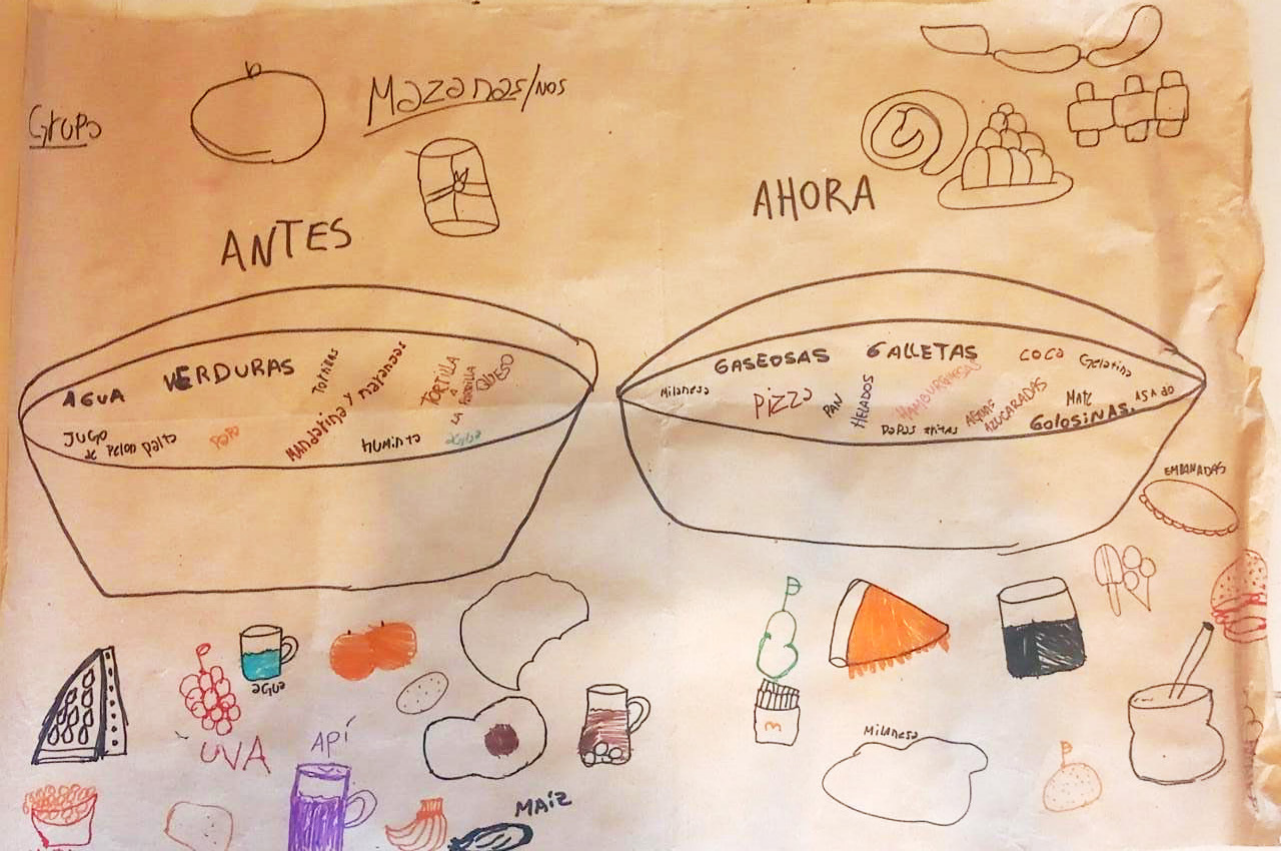
Ejemplo de “mapeo alimentario” realizado en una organización gremial de productores en Colonia Urquiza, La Plata, provincia de Buenos Aires, Argentina. Marzo, 2023.

La migración al cordón productivo platense implicó, para quienes llegaron, una serie de cambios, entre los que aparece el alimentario. Esto se expresa al analizar los resultados de la metodología de cartografía social que se ha desarrollado en el sector con un equipo más grande en el marco de talleres, denominada como “mapeos alimentarios”^42^. En los platos “de antes” no aparecían productos ultraprocesados ni comida rápida, sino preparaciones con alimentos frescos, muchas de ellas reconocidas por ser andinas o bolivianas, como sopas (maní, quinua, trigo, frangollo, arroz, choclo, fideos, maíz, pollo, vitina, verdura) o guisos (lenteja, quinua, trigo, papa lisa, arroz, fideos, mote, pollo), o jugos (soya, linaza, cebada). Al hablar de comidas actuales, en casi la totalidad de los mapeos se encontraban gaseosas, aguas saborizadas, pizza, hamburguesas, pan, papas fritas, por lo que gran parte del consumido registrado eran ultraprocesado. La mayor parte de los consumos actuales era de fideos, pan y arroz (Figura 4), aunque en los platos “actuales” también se registraba el consumo de comida o bebida casera (más de la mitad del total de los alimentos y bebidas registrados en los mapas eran caseros). 

Fuente: Fotografía tomada por la autora durante el trabajo de campo.

Esta diferencia era para Trifona (45 años), algo sabido previo a migrar a Argentina. Su hermano mayor, además de insistir que viajara a trabajar con él, le había advertido: “Él me dijo antes, allá no vas a probar nada de tu comida, allá es todo con pan y nada más”. haciendo alusión a la fuerte presencia de harina de trigo en casi todas las preparaciones que se consumen (Entrevista Trifona, marzo 2024). En su trabajo de campo, Castro^58^, retoma un chiste que parece alusivo, por el cual un hombre se jactaba que en Argentina “*sembraba fideos y arroz*”*.* Una investigación reciente sobre los consumos alimentarios en asentamientos de la Ciudad de Buenos Aires^59^ da cuenta de la cantidad de harina refinada consumida, siendo incluso identidad de quienes habitan; de hecho, la investigación se titula “*Somos de harina*”. 

Sin embargo, esto refiere a una transformación alimentaria de los migrantes de tan solo tres o cuatro décadas, en un contexto donde lo que se produce es, justamente, alimentos. Este cambio se expresaba en los adultos y pronunciadamente en los niños, como primera generación familiar totalmente criada en Argentina, donde el consumo de alimentos frescos o bolivianos era reconocido por ellos como reducido, y se había generalizado la preferencia por ultraprocesados o comida rápida.

*Es que los niños te piden lo que ven en la escuela o en la publicidad, y cuando le damos comida nuestra no les gusta o les cae mal porque no están acostumbrados, ellos quieren chizitos, golosinas, papitas, hamburguesas, pizza, todo así*. (Registro de campo, diciembre 2022)

*Los chicos se acostumbraron a lo que comen en la escuela, milanesa, papa aplastada, arroz hervido con pollo; si vos les haces otra cosa no le van a comer, la comida boliviana no les gusta.* (Entrevista Trifona, marzo 2024)

Estos registros dan cuenta que la preferencia o rechazo a ciertos alimentos genera incluso expresiones biológicas como el “caer mal”. Investigaciones en el campo de la alimentación, han dado cuenta que, en ocasiones, estas expresiones fisiológicas, pueden ser inducidas por elementos no nutricionales ni bromatológicos, sino propios del significado de lo que se come^60^. 

Además, el deseo, gusto y la costumbre parecían ser las únicas explicaciones manifestadas por las interlocutoras. En este punto, es útil señalar que las preguntas sobre la alimentación no deberían quedarse únicamente con explicaciones racionales y formuladas conscientemente en entrevistas. Como expresó una productora: “*No podés guiarte por lo que te dicen, ellos te van a decir que comen lo que producen, pero no es así*” (Registro de campo, febrero 2024). La riqueza de la etnografía es que permite profundizar en el entendimiento de estas preferencias y rechazos, desde un marco histórico más amplio, tensionando lo verbalizado de lo practicado. ¿Qué otras claves pueden ayudar a pensar, además de las explicitadas como la costumbre o el deseo, la transformación alimentaria? 

Tres cuestiones centrales. Primeramente, la accesibilidad económica, productiva y espacial. Esto atañe al pasaje de economías desde los lugares de origen, donde gran parte de lo consumido era autoproducido, a la prevaleciente en el cordón platense, donde se producen pocas variedades que se cultivan para el mercado.

*Antes se tenía la propia producción, los abuelos tenían su quinta y comían de ahí, no necesitaban de alguien más, acá ya no es así, tenes que depender del almacén.* (Registro de campo, junio 2023)

El consumo alimentario no puede separarse, en ningún grupo social, de su posibilidad de acceso. Debemos detenernos en la situación de vulnerabilización mencionada, y en una de las características principales de las unidades de producción del cordón: la contracción del consumo. Al ser los ingresos insuficientes, estos se utilizan para acceder a alimentos rendidores de menor costo y calidad, y alimentos feculentos^61^. Patricia Aguirre^62^^,^^63^, ha analizado profusamente cómo la crisis económica en Argentina, desde 1965 hasta 2000, dividió el patrón alimentario previamente unificado, en comida “de ricos” y comida “de pobres”. Al existir bajos ingresos, los alimentos deben cumplir principalmente la función de saciar, acción que hacen por su contenido graso, lo cual hasta los panificados pueden portar. Entre estos grupos, el principio de incorporación que rige es que sean alimentos “rendidores”, es decir, “baratos”, “que llenen” y “que gusten”^62^. 

Esta baja accesibilidad económica deja muchas veces por fuera incluso productos empaquetados, que se constituyen como objetos de deseabilidad más no de fácil adquisición:

*Los niños comentaron que lo más usual era levantarse y tomar té con pan, a veces licuado de alguna fruta, si se podía pan casero. Cuando pregunté sobre las galletas en paquete, expresaron que “solo cuando hay dinero”, como las gaseosas, o golosinas. Se comparaban con chicos de su escuela que consumen mucho las papas fritas del centro, pero ellos pocas veces, aunque les gusta, pues viven muy lejos de donde se venden.* (Registro de campo, noviembre 2022)

En este fragmento, el “*cuando se puede*” delimita la accesibilidad tanto económica como espacial (vivir lejos) de ciertos consumos. Además, como registran Lemmi y Muscio^36^, debemos incorporar al registro la vulnerabilidad asociada a la falta de un espacio físico para la preparación de alimentos, de cocina/horno, y electrodomésticos ahorradores de tiempo. 

En segundo lugar, una cuestión que se reiteraba en el trabajo de campo era la falta de tiempo para comer bien. Para pensar la temporalidad y su implicancia en la alimentación, debemos apuntar que, en este cordón, las tareas reproductivas como la cocina, se realizan casi en paralelo a las productivas, mientras que las largas jornadas laborales reducen el tiempo disponible para realizar preparaciones. Esto lleva a que se consuman menos comidas (el almuerzo o la cena), salteando otras, o evitando platos que requieran mayor tiempo de elaboración. La “falta de tiempo” en los cordones hortícolas equivale necesariamente a hablar de los tiempos del mercado, aquellos que establece el modelo al disponer la demanda de alimentos, así como la velocidad con que la logística comercial debe sacar la producción de las quintas. A su vez, estas tareas son llevadas a cabo casi en su integralidad por mujeres y el hecho de que la unidad productiva y doméstica coincidan en el mismo predio, y que todo el grupo familiar aporte para realizar la producción lleva a que las tareas reproductivas queden desdibujadas, dado que se trata de un trabajo no contabilizado ni remunerado^36^^,^^52^. Esto resulta central, dado que la sobreexplotación de las mujeres en la esfera de la reproducción social y al interior de las unidades domésticas productivas, en especial las alimentarias, asegura la reproducción de la fuerza de trabajo, así como la producción de alimentos frescos^53^. 

Estos puntos son condicionamientos que transforman los consumos alimentarios y son centrales, pero no únicos. Si no, ¿cómo podría explicarse el desecho o rechazo de las legumbres, aun cuando su disponibilidad estuviera garantizada en la entrega de bolsones escolares, y siendo este grupo de alimentos acervo alimentario de la comida boliviana?

*Un productor comentó que antes él tomaba sopa de lentejas de desayuno y que ahora no se come tanto, en su casa viene en el bolsón y no se consumen.* (Registro de campo, julio 2023)

*“Hay que comer más legumbres”, escuché mientras comían las ensaladas que habíamos preparado, y se comenzó a dialogar cómo antes se consumía legumbres en todos los platos y progresivamente se fue dejando.* (Registro de campo, mayo 2023)

Por eso, el tercer catalizador que interesa resaltar en las transformaciones alimentarias se vincula con el doble juego de desvalorización y valorización alimentaria. En una escena dentro de una oficina pública con una productora “*se notó la forma en que ella bajó abruptamente su tono de voz al decir que su madre cultivaba alimentos como chirimoya, oca, papa lisa, y cazaba palomas cuando no había para comer*” (Registro de campo, mayo 2022). Si bien se consideró que sería algo ocasional, el trabajo de campo fue mostrando lo que parecía un nudo de la cuestión. Un día, Tomasa^43^, habló explícitamente de esta discriminación:

Tomasa: *Te lo dicen directamente, “yo no comería mote, porque eso es para las gallinas”. Saben decir… ahí cuando vos los invitas.* [...] I*ncluso algunos bolivianos, mi hija tenía un compañero que era hijo de bolivianos, y él discriminaba a los mismos bolivianos, a los chicos les decía bolivianos y se reía de lo que traían para comer, porque él llevaba galletitas o golosinas.*

Autora: ¿*Aunque sean todos paisanos en la escuela?*

Tomasa: *Es que no hay escuelas solo de paisanos, mezclado está, pero se sufre mucha discriminación por eso, algunos papás que entienden esto se van a Bolivia, por los chicos más que nada. Yo le decía “respondele: comida boliviana con orgullo”.* (Entrevista Tomasa, marzo 2024)

Lo interesante del relato es que ella ha sido promotora de alimentación en los talleres alimentarios mencionados, y es usual que resalte lo que aprendió al formarse: la revalorización de su comida. Como ha dicho en los talleres “*queremos que se sientan orgullos compañeros, de las preparaciones que ustedes conocen, y les da vergüenza o no se animan a contar*” (Registro de campo, septiembre 2023). 

La desvalorización de la comida reconocida como boliviana iba acompañada de la valorización de lo argentino, sociedad receptora, asociada con el fetiche del prestigio, la modernidad y el progreso. A raíz del análisis etnográfico digital, se registró el orgullo denotado en redes sociales por comida ultraprocesada y rápida, como hamburguesas, papas fritas, pizzas o sándwiches de milanesa (como se presentó al comenzar este apartado, no se registraba en sus lugares previos a migrar). Esto mismo fue señalado por Elodia (49 años), durante el transcurso de un taller alimentario:

*A mí me llama la atención que ustedes, compañeros, suban con orgullo a sus estados fotos cuando van a McDonald ‘s y no de la sopa que aprendieron en su casa. ¿Por qué hacen eso, si saben que esa hamburguesa les va a hacer mal y no es algo nuestro?* (Registro de campo, mayo 2023)

Otra productora comentó: “*A mí la nutricionista me armó un plan, saca comidas pesadas y pone livianas, yogures, frutas”* (Registro de campo, septiembre 2023). Dentro de los valores de los alimentos, el lenguaje nutricional es central. En el trabajo de campo, es habitual encontrar niños consumiendo yogures, los cuales se reservan para los más pequeños, porque son productos más costosos^64^, siendo que existe un conocimiento que relaciona lácteos, como yogures o leche, con alimentos sanos para el crecimiento. En algunos talleres, al medir el azúcar que contienen estos productos, se logró escuchar “*no puedo creer que lo que me dijeron que era para que* é*l crezca en verdad lo llena de azúcar*” (Registro de campo, diciembre 2022). 

**Figura 5:**
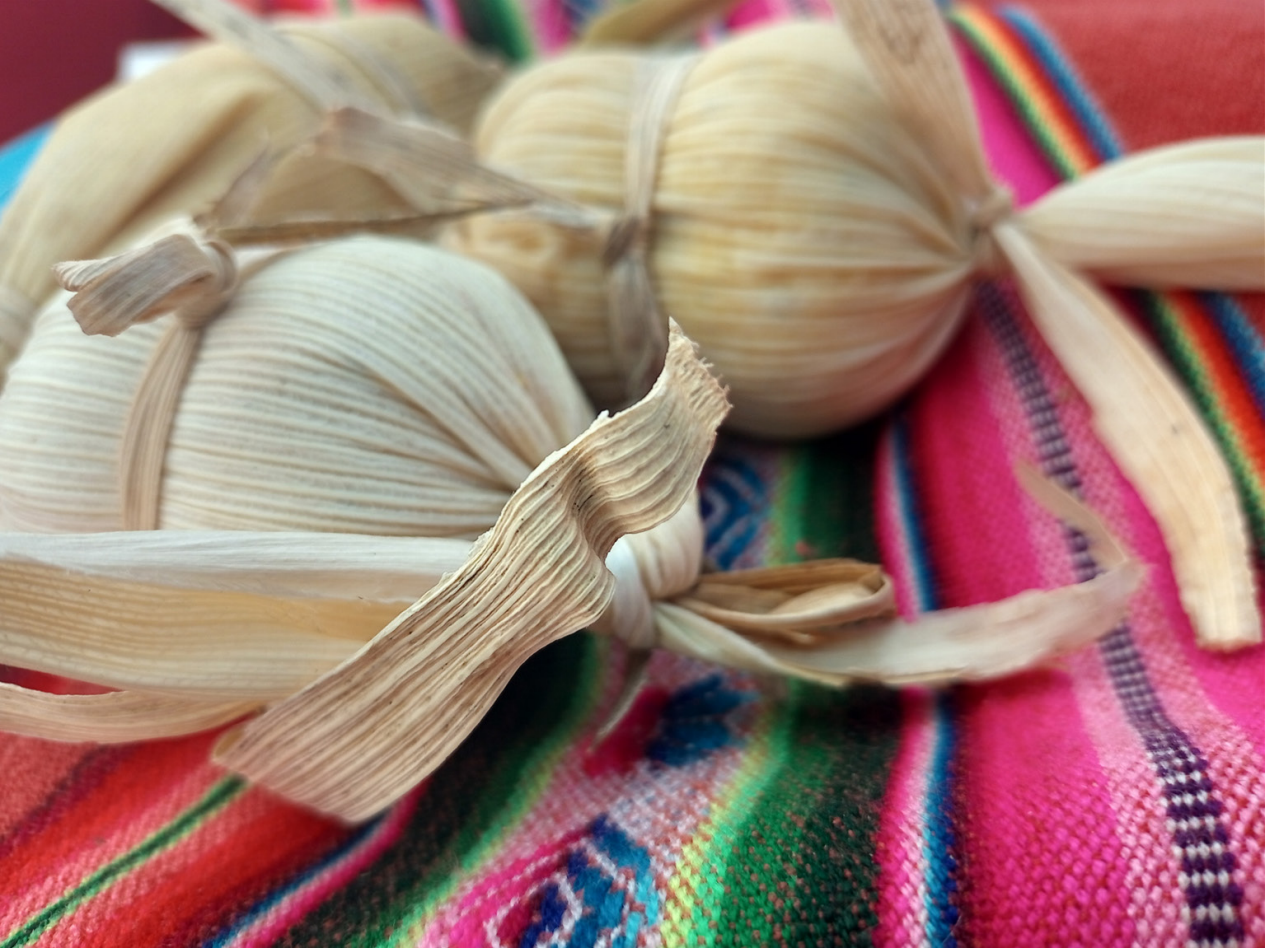
Tamales caseros con ingredientes de autoproducción realizados en Abasto, La Plata, provincia de Buenos Aires, Argentina. Septiembre, 2022.

**Figura 6:**
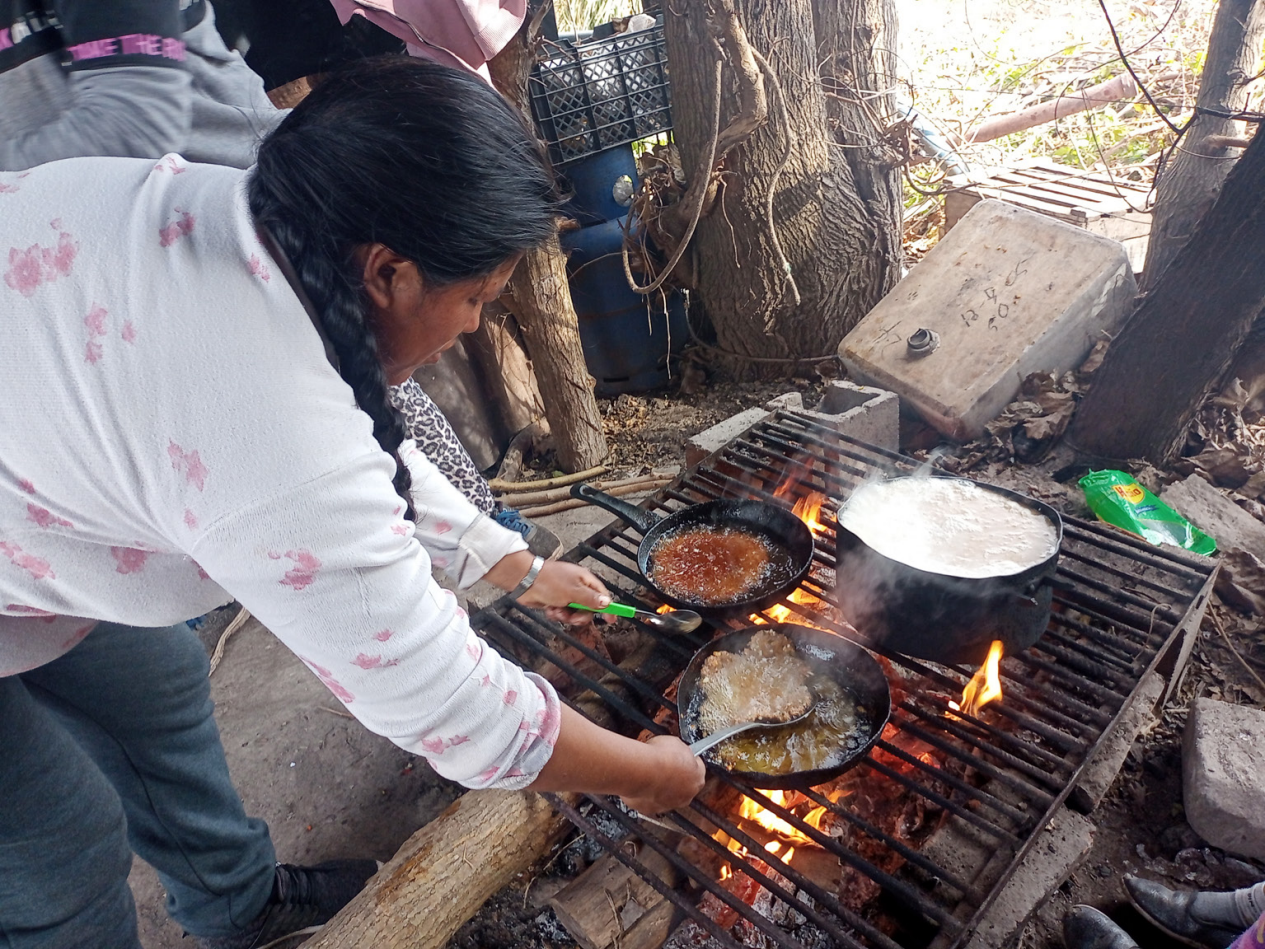
Preparación de sopaipillas y arroz graneado en Abasto, La Plata, provincia de Buenos Aires, Argentina. Abril, 2022.

Estas lecturas que asociaban lo boliviano con lo indeseable y lo argentino con lo deseable no eran lineales, sino que los matices eran parte de la alimentación en el cordón. Por ejemplo, se resaltaba la vinculación de las comidas bolivianas con sentimientos de añoranza y nostalgia. Por redes sociales, eventualmente, se compartía la comida boliviana y el recuerdo de quienes las cocinaban: “*la mamita*” o “*la abuelita*”. Esto se alineaba con el importante proceso de escalada de comedores paisanos y de legumbrerías ya mencionado, que según Tomasa, “¡*ni siquiera en Tarija hay tantos!*” (Registro de campo, abril 2024). Además, en ocasiones festivas como el Día de Todos los Santos, el 1° de agosto o en Carnavales, se compartían especialmente fotos en las redes sociales (estados de WhatsApp y en grupos) de recetas como tantawawas (panes que son ofrendas), chicha morada o tamales (Figura 5 y Figura 6). 

Fuente: Fotografía tomada por la autora durante el trabajo de campo.

Fuente: Fotografía tomada por la autora durante el trabajo de campo.

Se expresa así la conformación y expansión de un “nosotros alimentario”, en un contexto que tiene características segregatorias por parte del país receptor; un “nosotros alimentario” que coexiste y se retroalimenta recíprocamente con el deseo de inclusión en dicha sociedad y sus marcas de consumo. 

Caracterizar la valorización y desvalorización alimentaria requiere enmarcarlo en un proceso mayor por el que, desde el periodo colonial, muchas de las tensiones sociales se manifestaron en clasificaciones y jerarquizaciones que se hicieron de las cocinas, las dietas y los alimentos nativos; la colonización inculcó que algunos alimentos sean concebidos como sucios, feos, inferiores e incivilizados^10^, marginándolos como sucedió con la quinua y el amaranto, y su reemplazo por especies europeas^65^. 

En esta investigación, ciertos productos ultraprocesados tenían una valoración simbólica alta para quienes se encontraban por fuera de los parámetros de una sociedad blanqueada y europeizada como la argentina, aunque como se señaló, no era posible consumirlos en la cantidad deseada debido a su costo. Astelarra^66^ sostiene que el deseo de los productores bolivianos de migrar estuvo y está vinculado a su inclusión en las instituciones de la modernidad: en el terreno productivo, la “erosión cultural” implicaba la necesidad de incorporar directa y rápidamente los valores de la sociedad moderna y urbana como estrategia productiva para sobrevivir en el cordón; en el terreno del consumo, esa necesidad de incorporación no fue tan lineal ni rápida, pero sí pronunciada, en el marco de relaciones de desigualdad. 

¿Por qué se expresa que no fue ni rápida ni lineal? Si bien se registra una transformación en favor de alimentos altos en harinas refinadas, y el deseo de adquisición de ultraprocesados y comida rápida de la sociedad receptora, concordantemente, se acrecienta el número de comedores paisanos y legumbrerías, junto con la asociación entre la comida boliviana y la familia, la añoranza y la nostalgia. Por eso, como elabora García Canclini^67^, los alimentos, como todo elemento que conforma la identidad de una cultura, nunca se pierde por completo, sino que se resguardan. 

Para finalizar este apartado, Margulis^68^ señala que el racismo no reside únicamente en el señalamiento o clasificación de las diferencias, sino también en la demarcación y negación de lo diferente, leído desde escalas sociales jerarquizadas que se estructuran sobre lo bueno/malo, sano/insano; rico/no rico liviano/pesado. El racismo se expresa también en la negación, el encubrimiento y el silenciamiento de las preparaciones que los migrantes recrean, así como en la sobrevaloración de los alimentos ultraprocesados e industriales que abundan en el país receptor, asociado con nociones de modernidad, progreso y diversión. 

No se trata de un ejercicio racista lineal desde la sociedad receptora hacia los migrantes, sino que, al estar anclado en alimentos y bebidas en sí mismas, son valorizaciones interiorizadas por los mismos sujetos, practicadas entre paisanos. A partir de esto, se reitera la importancia de considerar el dinamismo que ha tenido la expresión del racismo, la cual no se circunscribe únicamente a la asociación con el color de piel^24^, ya que, por distintos procesos de negación e invaloración, las preparaciones y consumos alimentarios también pueden portar racismo. 

## DISCUSIÓN

Una pregunta que surge al caracterizar el cordón productivo del Gran La Plata es ¿por qué los migrantes permanecen realizando esta tarea, aún en condiciones tan adversas? Me precede una larga y valiosa discusión sobre la persistencia de las unidades agrícolas de trabajo familiar en contextos capitalistas como el cordón platense. Desde algunos argumentos, se ha enfatizado la brecha entre tiempo de trabajo y tiempo total de producción, que hace que el capital, al no conseguir dominar integralmente el tiempo de producción de la mercancía, desaliente su participación en esta actividad^69^. Otras lecturas señalan la explicación en las propias particularidades de las unidades de producción familiares, que se contentarían eventualmente con la reproducción de las necesidades familiares y de las condiciones de producción, y así persistirían en la actividad^54^. 

En este trabajo se considera que ambas posturas no son excluyentes, sino confluyentes y que, para el caso en cuestión, otro argumento precisaría ser profundizado: el racismo. Como se presentó a lo largo de este escrito, el racismo se expresa como mecanismo de anclaje de los productores en la actividad, al subordinarlos y descatalizar la movilización a otras actividades. Esto opera, claro está, en un mercado laboral argentino que, siendo históricamente receptor de migrantes limítrofes, los ha clasificado, atribuyéndoles inferioridad racial; discriminación negativa que segmenta los mercados de trabajo y les asigna los nichos laborales más desfavorables^70^. En la historia nacional, los migrantes limítrofes han tendido a ocupar puestos laborales en trabajos que los nativos argentinos no aceptaban, superando el déficit de mano de obra^26^. Siguiendo con Pizarro^55^^,^^71^^,^^72^, la segmentación étnica implica el acceso diferenciado de los inmigrantes al mercado laboral. Allí operan mecanismos de discriminación basados en la marcación de la alteridad étnica y/o racial, que permiten que estos estereotipos y prejuicios raciales justifiquen la ubicación de los trabajadores bolivianos en las posiciones inferiores de la jerarquía laboral^72^. 

En el cordón platense, si bien es reconocido que los productores bolivianos son el sector más pobre y menos capitalizado^37^, con lo dicho en estas líneas interesa señalar que esto se intersecta con el proceso de racialización como población migrante en el cordón platense. Para ello, resulta iluminadora la categoría que utiliza Margulis^68^, de *racialización de las clases sociales*, porque en la Argentina blanca, en el lenguaje ordinario, lo “negro” se tiende a asociar a los “pobres”^26^. Se trata, entonces, de la constitución histórica de una dinámica de desigualdad de clase y racial en el seno mismo de la estructura y producción del cordón. Además, se intersecta con un proceso de sobreexplotación y subordinación de las mujeres, en el cual se invisibilizan y desvalorizan las tareas productivas y reproductivas que ellas llevan adelante, que permiten la existencia del sector y la producción de alimentos. 

Sin embargo, el modelo racista de producción de alimentos y su penetración en los consumos alimentarios, no es totalizador de las formas de existencia en el cordón. La escalada de comedores paisanos y legumbrerías denota la posibilidad de reapropiación y resignificación de la comida boliviana, reformulación del “nosotros alimentario”, que posibilita la existencia en el espacio, como lugar deseado. 

## PALABRAS FINALES

Las cifras alarmantes que se presentan al inicio del texto dan cuenta del problema estructural del hambre y la malnutrición en Argentina. Buscando ampliar la mirada, la pregunta por el racismo en los procesos alimentarios motorizó este escrito, recortando a un contexto particular: el cordón productivo más capitalizado de Argentina, ubicado en el Gran La Plata. 

En las líneas trabajadas, se encontró que ciertas dinámicas racistas se expresan en la configuración del cordón platense, en la producción y los consumos, dinámicas que no son lineales, únicamente desde la sociedad receptora a la migrante, sino que se propagan por todo el tejido social, incluso ejercidas por los productores migrantes. 

En el eje productivo, la génesis misma del cordón, desde el segundo período migratorio, estuvo conformada por el ejercicio racista, que quedó anclado en las memorias de quienes hoy trabajan allí (esclavización de niños, abusos a adultos analfabetos, violencia sexual contra las niñas, etc.). Hecho no anecdótico, puesto que se recuperan continuidades en la actualidad con esas prácticas. Además, se retomó la cotidianeidad en el cordón atravesada por la discriminación negativa. 

En el eje del consumo de preparaciones, las transformaciones de la alimentación de las personas migrantes al llegar al cordón hortícola pueden explicarse tanto por la contracción del consumo, debido a los fluctuantes y bajos ingresos; a la disponibilidad del tiempo de mercado; y a las valorizaciones asociadas con la comida “argentina” y “boliviana”, ligadas a la importancia de integrarse. Si la transformación productiva fue condición de posibilidad para existir en el cordón del Gran La Plata, en la esfera alimentaria, su transformación implicó una serie de valorizaciones y desvalorizaciones entre la comida argentina y boliviana. Se detuvo en las líneas precedentes especialmente en el mecanismo de resguardo, que configuró un proceso novedoso y necesario de resaltar: la escalada de comedores paisanos y legumbrerías, que denotan la posibilidad de reapropiación y resignificación del “nosotros alimentario”. Esta recreación de la identidad, aún mayor que en sus lugares de origen, debe entenderse como mecanismo creativo de anclaje en la sociedad receptora, que opera en la permanencia de estos colectivos migrantes en el trabajo productivo, convirtiéndolo en lugar de deseabilidad para ellos. 

En todos estos procesos, las mujeres se integran como encargadas y aseguradoras de la reproducción social, protagonizando los procesos alimentarios de las familias productoras. Esto conlleva dinámicas de sobreexplotación de las mujeres al interior de las unidades domésticas productivas, la cual asegura la reproducción de la fuerza de trabajo, así como la producción de alimentos frescos y flores de corte.

Se espera haber argumentado por qué no es un capricho teorizador nombrar que nuestras preparaciones y consumos alimentarios portan racismo, un racismo que ni siquiera necesita mecanismos externos, porque las mismas personas racializadas lo han internalizado y lo esparcen. Al decir de Rita Segato, entendiendo el racismo como *verdaderos silencios cognitivos e indiferencia etnográfica* que permiten afirmar que al continente (y a las discusiones sobre las preparaciones y cocinas) les cuesta considerar del color de la piel, se finaliza destacando que la categoría permite también complejizar los procesos de transformación alimentaria, alertando que cualesquiera sean las salidas proyectadas, deberán considerar esta realidad racializada.
